# A novel assay of thrombotic risk

**DOI:** 10.1186/1753-6561-7-S1-O4

**Published:** 2013-01-30

**Authors:** Fahad Buskandar, M Alobaidly, N Moran

**Affiliations:** 1 Molecular & Cellular Therapeutics, Royal College of Surgeons in Ireland, Dublin, Ireland

## Introduction

It is widely believed that platelet responsiveness can be used as a marker for assessing thrombotic risk in patients. Therefore, there have been many attempts to develop suitable assays for quantifying platelet responses in clinical samples 1. Platelet aggregometry is widely used, but is limited in its ability to assess platelet hyper-responsiveness. In order to develop a better assay of thrombotic risk, we use a novel assay to evaluate platelet secretion of adenosine triphosphate & diphosphate (ATP/ADP) in response to various agonists. This assay measures both the maximal amount of adenine nucleotides released by a range of platelet activators and the potency of each activator.

## Methods

To determine the reproducibility of this assay, we assessed 4 healthy female subjects on 3 separate occasions. 10mls of blood was drawn from subjects who had abstained from medication for the previous 12 days. Platelet ATP/ADP secretion was assessed in a 96 well assays as previously described 2. Briefly, platelet secretion is assessed in response to increasing doses of platelet agonists (Thrombin receptor activating peptide: TRAP 0.1-32µM; Collagen related peptide: CRP 0.05-100µg/ml). Released ATP/ADP is measured using firefly luciferase (Chronolume Corp). Data are expressed as nmoles ATP/ADP secreted per 10^6^ platelets. Dose-response curves are constructed and analysed using GraphPad Prism 5.0.

## Results

The maximal amount of ATP/ADP released is similar for both agonists tested (1.94±0.25 and 2.12±0.28 nmoles per 10^6^ platelets in response to TRAP and CRP, respectively). However, the potency of responses, measured as EC_50_ values, differed for the two agonists. For TRAP, the EC_50_ values were equivalent in all 4 donors (mean EC_50_ value is 4.84 ±0.30µM; range 4.38-5.57µM). in response to CRP, the potency of the responses were nearly similar for all the donors (EC_50_ range from 0.37µg/ml to 1.08µg/ml). Nonetheless, there is a high degree of concordance within all samples from any one donor.

## Conclusion

Our data demonstrate that individual donors display unique response-parameters which may be used to assess thrombotic risk. In addition, we can conclude that the dose of agonist that causes a half-maximal response is a reliable index of platelet responsiveness.
